# The inhibitory effect and mechanism of quetiapine on tumor progression in hepatocellular carcinoma in vivo

**DOI:** 10.1002/tox.23380

**Published:** 2021-10-09

**Authors:** Chun‐Min Su, Song‐Shei Lin, Hsiao‐Chia Wang, Fei‐Ting Hsu, Jing Gung Chung, Li‐Cho Hsu

**Affiliations:** ^1^ Department of Surgery Show Chwan Memorial Hospital Changhua Taiwan, ROC; ^2^ Department of Medical Imaging and Radiological Sciences Central Taiwan University of Science and Technology Taichung Taiwan, ROC; ^3^ Emergency Department Cathay General Hospital Taipei Taiwan, ROC; ^4^ School of Medicine Fu Jen Catholic University New Taipei City Taiwan, ROC; ^5^ Department of Biological Science and Technology China Medical University Taichung Taiwan, ROC; ^6^ School of Medicine National Yang‐Ming Chiao‐Tung University Hospital Taipei Taiwan, ROC

**Keywords:** ERK, hepatocellular carcinoma, NF‐κB, quetiapine

## Abstract

Hepatocellular carcinoma (HCC) is the primary tumor of the liver and the fourth leading cause of cancer‐related death. Recently, several studies indicated the anti‐tumor potential of antipsychotic medicine. Quetiapine, an atypical antipsychotic, is used to treat schizophrenia, bipolar disorder, and major depressive disorder since 1997. However, whether quetiapine may show potential to suppress HCC progression and its underlying mechanism is persisting unclear. Quetiapine has been shown to induce apoptosis and inhibit invasion ability in HCC in vitro. Here, we established two different HCC (Hep3B, SK‐Hep1) bearing animals to identify the treatment efficacy of quetiapine. Tumor growth, signaling transduction, and normal tissue pathology after quetiapine treatment were validated by caliper, bioluminescence image, immunohistochemistry (IHC), and hematoxylin and eosin staining, respectively. Quetiapine suppressed HCC progression in a dose‐dependent manner. Extracellular signal‐regulated kinases (ERKs) and Nuclear factor‐κB (NF‐κB) mediated downstream proteins, such as myeloid leukemia cell differentiation protein (MCL‐1), cellular FLICE‐inhibitory protein (C‐FLIP), X‐linked inhibitor of apoptosis protein (XIAP), Cyclin‐D1, matrix metallopeptidase 9 (MMP‐9), vascular endothelial growth factor‐A (VEGF‐A) and indoleamine 2,3‐dioxygenase (IDO) which involved in proliferation, survival, angiogenesis, invasion and anti‐tumor immunity were all decreased by quetiapine. In addition, extrinsic/intrinsic caspase‐dependent and caspase‐independent pathways, including cleaved caspase‐3, −8, and − 9 were increased by quetiapine. In sum, the tumor inhibition that results from quetiapine may associate with ERK and NF‐κB inactivation.

## 1 INTRODUCTION

Antipsychotics, which effectively improve symptoms of bipolar disorder and schizophrenia, are divided into typical and atypical antipsychotics.^1^ It is worth noting that long‐term use of antipsychotic drugs associates with the reduction of cancer risk in patients with schizophrenia.^2^, ^3^ Furthermore, cell and animal models have been used to study the anti‐cancer property of antipsychotics. Antipsychotic has been shown to possess potent anti‐cancer properties which are involved in suppression of tumor growth, invasion, metastasis, and reversion of chemoresistance.^4^, ^5^, ^6^

For instance, typical antipsychotics such as thioridazine and trifluoperazine have been indicated leading osteosarcoma cancer stem cells to death through targeting autophagy^7^ as well as sensitizing bladder cancer to chemotherapy by inhibition of B‐cell lymphoma‐extra‐large (Bcl‐xL) expression, respectively.^8^ In addition, atypical antipsychotics such as risperidone and olanzapine have been demonstrated to trigger growth inhibition of colorectal and pancreatic cancer cells through induction of apoptosis and suppression of survivin expression.^3^, ^9^

Although numerous studies presented that antipsychotics show encouraging antitumor efficacy in cells and animal models, others provide tumor growth conflict evidence.^8^, ^10^, ^11^ Antipsychotics such as olanzapine (3 mg/kg/day) and quetiapine (25 mg/kg/day) have been found to correlate with increased risk of liver cancer and thyroid adenoma in mice.^11^ Previous studies indicated that suppression of extracellular signal‐regulated kinases (ERK)/protein kinase B (AKT) signaling and induction of apoptosis were associated with quetiapine‐inhibited cell survival and invasion in HCC in vitro.^4^ However, whether quetiapine influences the progression of HCC in vivo has not yet been elucidated. Therefore, the main purpose of the present study was to verify the effect and mechanism of quetiapine in HCC in vivo.

## 2 METHODS

### 2.1 Reagents and antibodies

Dimethyl sulfoxide (DMSO) was purchased from Sigma Chemical Co. (St. Louis, MO). Primary antibodies purchased from different companies were all listed as follows: PD‐L1 (Abcam, Cambridge, UK), IDO (Sino Biological, Beijing, China), MMP‐9, VEGF, and P‐ERK (EMD Millipore, Billerica, MA), XIAP, CyclinD1 (Thermo Fisher Scientific, Fremont, CA), MCL‐1 (BioVision, Milpitas, CA), NF‐κB p65 (Ser536), cleaved caspase‐3, cleaved caspase‐8, cleaved caspase‐9, C‐FLIP (Cell signaling Technology, Danvers, MA), ERK1/2(Thr202/Tyr204) (Elabscience Biotechnology Inc., Houston, TX), β‐actin (Santa Cruz, CA). Secondary antibodies were obtained by Jackson Immune Research (West Grove, PA).

### 2.2 Culture of Hep3B and SK‐Hep1 cells

Both human hepatocellular carcinoma (HCC) cells were maintained in Dulbecco's Modified Eagle's medium (DMEM) with 10% fetal bovine serum, 1% penicillin–streptomycin. All above reagents were purchased from Hyclone (GE Healthcare Life Sciences, Logan, UT) and Gibco (Life Technologies, Carlsbad, CA). Cells were incubated in 37°C humidified incubator with a 5% CO_2_ atmosphere.

### 2.3 Luciferase (luc2) reporter gene establishment in Hep3B and SK‐Hep1 cells

Both Hep3B cells and SK‐Hep1 were transfected with pGL4.50 (luc2/CMV) vector as previously described.^12^, ^13^ Cells with constitutive luc2 signal expression were selected by 200 μg/ml of hygromycin and named Hep3B/*luc2* and SK‐Hep1/*luc2* cells.

### 2.4 Animal experiment of Hep3B and SK‐Hep1 bearing mice

The animal experiment was approved by Institutional Animal Care and Use Committee in China Medical University, with certification number: CMUIACUC‐2020‐367. Six‐week‐old BALB/cAnN.Cg‐Foxn1^nu^/CrlNarl (nude mice) was purchased from the National Laboratory Animal Center, Taipei, Taiwan. Ten million SK‐Hep1 and Hep3B cells were subcutaneously injected into mice's left flank and allowed tumor progression for 14 days.^14^ After the tumor reached 100–120 mm^3^, mice were randomly divided into three treatment groups (*n* = 4–5/group). Three treatment groups for therapeutic efficacy evaluation have included, control (0.1% DMSO in 100 μl phosphate‐buffered saline buffer, PBS), quetiapine 10 mg/kg (dissolved in 100 μl PBS), and quetiapine 20 mg/kg (dissolved in 100 μl PBS). Mice tumor size was recorded and analyzed by following format (volume = length×width^2^×0.523).^15^ Mice body was recorded every 2 days. The tumor growth rate of each mouse was measured by followed formula: TGR (mm^3^/2 days) = (V_Day10_ − V_Day0_)/(10 days/2 days).^13^ Mice were finally anesthetized by overdose isoflurane (>3%) and then sacrificed on day 10. Tumors were extracted for photographed and performed with further staining procedure.

### 2.5 In vivo bioluminescence image of Hep3B and SK‐Hep1 bearing mice

D‐luciferin was intraperitoneally injected with 150 mg/kg D‐luciferin for 15 min before scanning. Mice were maintained in the chamber with 1%–3% isoflurane and imaged tumor by Xenogen IVIS imaging system 200 series (Xenogen, Alameda, CA) at an acquisition period of 10 s (*n* = 3). Luc signal from the tumor was quantified under photon/s/cm^2^/sr unit by Living Image version 2.20.

### 2.6 Hematoxylin and eosin stain of liver, spleen, kidney organs

Liver, spleen, kidney organs were extracted from mice and fixed by 10% neutral buffered formalin. Then, the tissue was embedded in paraffin and sliced for hematoxylin and eosin (H&E) and IHC staining. The embedded, sliced, and staining procedure of tissue was performed by Bond biotech, Inc. (Taichung, Taiwan). Finally, the pathology images of each group were captured by a light microscope (Nikon ECLIPSE Ti‐U, Minato City, Tokyo, Japan) at ×100.^16^

### 2.7 Immunohistochemistry stain of Hep3B and SK‐Hep1 tumor tissue

Tumor tissues were extracted from mice and fixed by 10% neutral buffered formalin. Embedded and sliced were conducted by Bond biotech, Inc. as well. Immunohistochemistry (IHC) staining procedure was followed by Tsai et al.'s study.^17^ Quantification of IHC images were performed by using ImageJ with IHC Image Analysis Toolbox. At least five views from each group of IHC images were calculated.

### 2.8 Statistical analysis

All quantitative data were presented as the mean ± *SD* from three independent experiments. One‐way analysis of variance was used to compare the control and quetiapine‐treated groups. The *p*‐value smaller than .05 was defined as a significant difference between the control and quetiapine‐treated groups.

## 3 RESULTS

### 3.1 Quetiapine effectively suppressed Hep3B and SK‐Hep1 tumor progression

**FIGURE 1:**
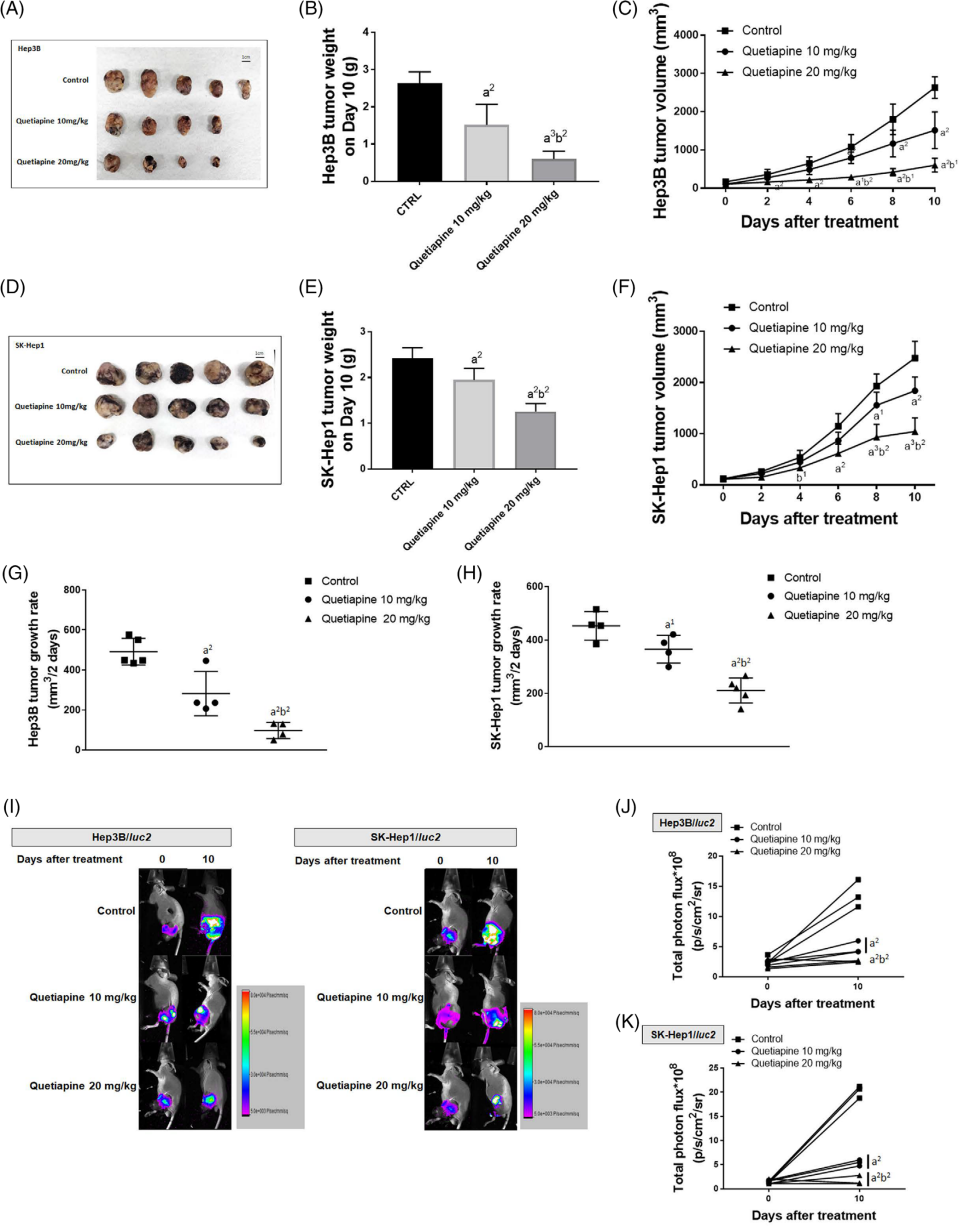
Hep3B and SK‐Hep1 tumor progression were found in the quetiapine‐treated group. (A, D) Tumor images on day 10, (B, E) average tumor weight, and (C, F) tumor volume from day 0 to 10 of each group are displayed. (G, H) The tumor growth rate of each mouse is displayed. (I–K) Luminescence images and quantification intensities of mice from the different groups on day 0 and day 10 are shown. (a^2^ *p*‐value <.01 vs. control; b^2^ *p*‐value <.01 vs. 10 mg/kg quetiapine)

To investigate whether quetiapine may have the potential to suppress HCC progression, we established Hep3B and SK‐Hep1 bearing model. As indicated in Figure 1A,B, the smallest Hep3B tumor on day 10 was found in the 20 mg/kg quetiapine treated group. Hep3B tumor inhibition potential was found in the 20 mg/kg quetiapine group since day2 after treatment (Figure 1C). Moreover, tumor inhibition results of quetiapine were also validated in another SK‐Hep1 bearing model. Markedly SK‐Hep1 tumor size reduction was found after 10 and 20 mg/kg quetiapine treatment as compared with the control group (Figure 1D,E,F). The tumor progression rate was found to be effectively suppressed by 10 and 20 mg/kg quetiapine in both Hep3B and SK‐Hep1 bearing models (Figure 1G,H). To further investigate whether the viable cells within the tumor region may decrease after quetiapine treatment, we established Hep3B/*luc2* and SK‐Hep1/*luc2* bearing model. The intensity of the luminescence signal may represent a living cells signal. In Figure I‐K, the signal intensity of Hep3B and SK‐Hep1 tumors were both suppressed by quetiapine therapy. Figure 1I‐K  illustrated the lower signal intensity under 10 and 20 mg/kg quetiapine administration conditions as compared to control. These results suggested that quetiapine might effectively suppress HCC progression.

### 3.2 Quetiapine suppressed anti‐apoptosis, proliferation, and metastasis‐related proteins were associated with inactivation of ERK/NF‐κB

**FIGURE 2:**
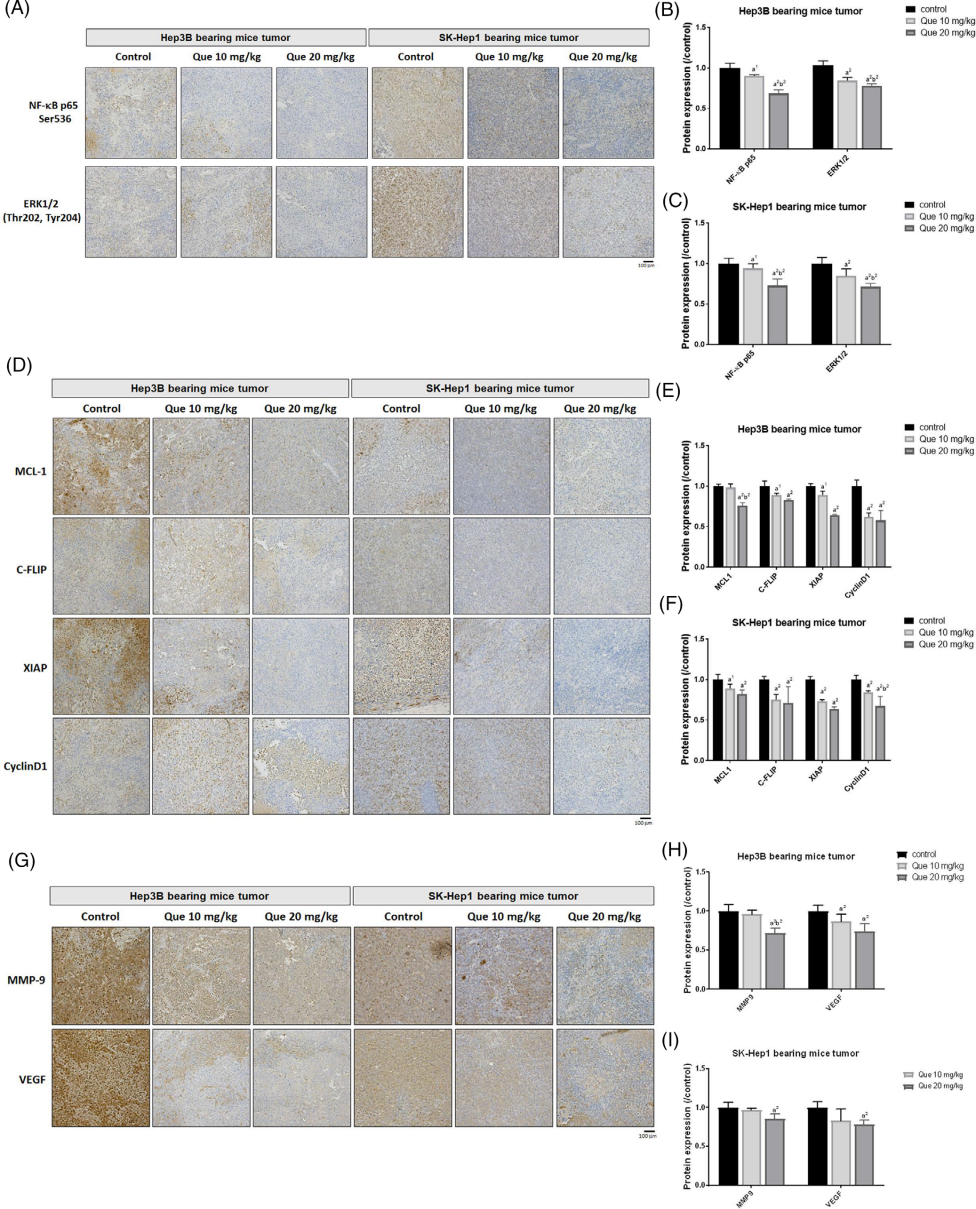
The inhibition of anti‐apoptosis, proliferation, and metastasis proteins expression of quetiapine may correlate to ERK/NF‐κB signaling inactivation. (A) Represented IHC staining images of phosphorylated ERK and NF‐κB, and their (B, C) quantification bar chart of each group are displayed. (D) Represented IHC staining images of MCL‐1, C‐FLIP, XIAP, cyclinD1, and their (E, F) quantification bar chart of each group are displayed. (G) Represented IHC staining images of phosphorylated MMP‐9 and VEGF, and their (H, I) quantification bar chart of each group are displayed. (a^1^ *p*‐value <.05, a^2^ *p*‐value <.01 vs. control; b^2^ *p*‐value <.01 vs. 10 mg/kg quetiapine)

In Figure 2A–C, quetiapine markedly suppressed the phosphorylation protein expression of ERK and NF‐κB. The quantification of IHC results suggested that 20 mg/kg of quetiapine showed superior protein level suppression as compared to other groups. The ERK/NF‐κB downstream protein that involved in anti‐apoptosis and proliferation,^18^, ^19^, ^20^, ^21^ such as MCL‐1, C‐FLIP, XIAP, and cyclinD1 was also decreased by quetiapine (Figure 2D). Quetiapine may suppress 0.5–0.9 times of MCL‐1, C‐FLIP, XIAP, and cyclinD1 protein expression level, the 20 mg/kg of quetiapine showed greater inhibition ability (Figure 2E,F). Additionally, metastasis‐related proteins; including MMP‐9 and VEGF were also decreased by 20 mg/kg quetiapine treatment about 0.6–0.9 times (Figure 2G–I). In sum, tumor anti‐apoptosis, proliferation, and metastasis‐related proteins were decreased by quetiapine.

### 3.3 Quetiapine activated the protein expression of apoptosis related proteins and suppressed the protein expression of immunosuppressive proteins in Hep3B and SK‐Hep1 bearing model

**FIGURE 3:**
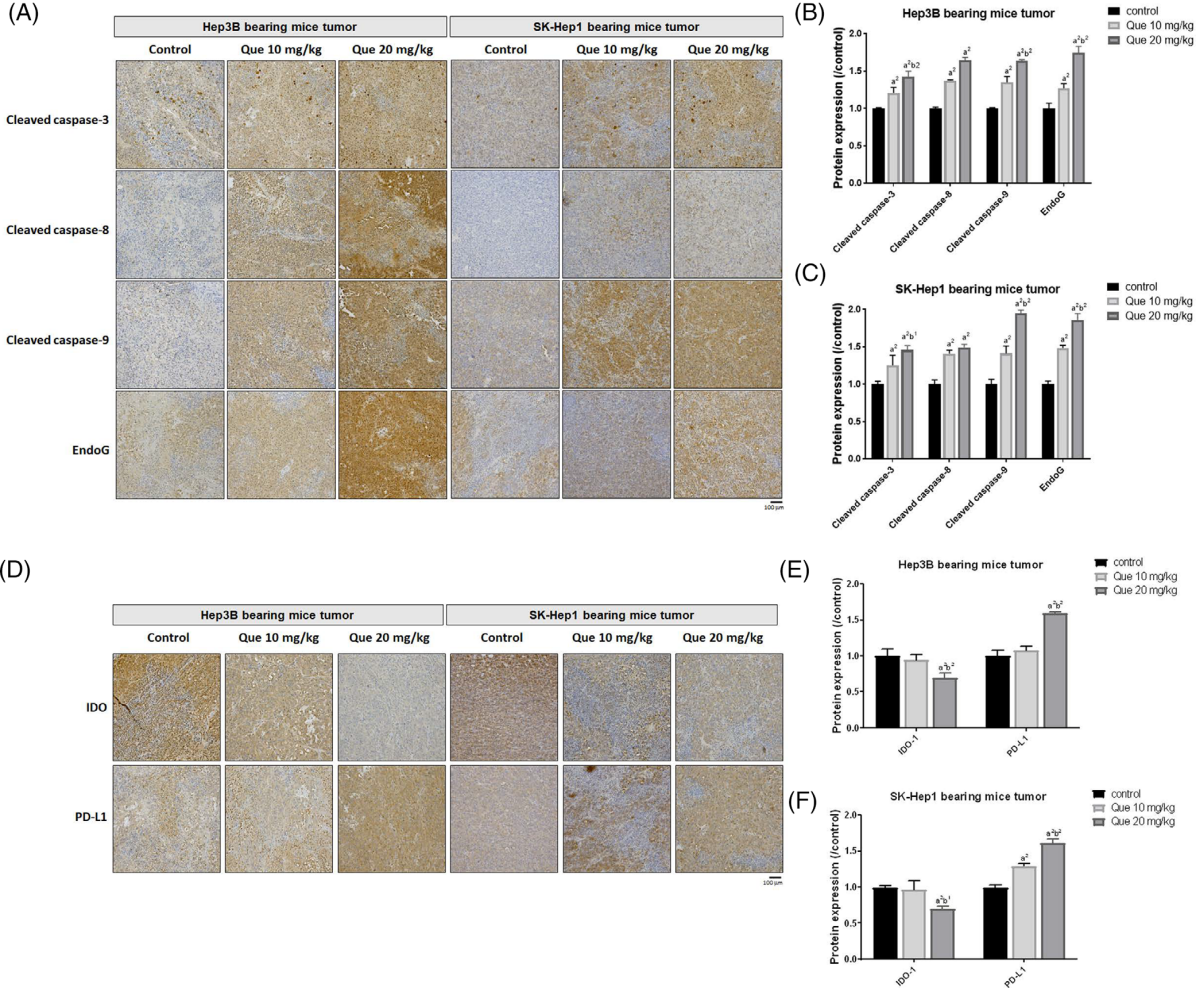
The induction of apoptosis and inhibition of immunosuppressive related proteins of quetiapine were found. (A) Represented IHC staining images of cleaved caspase‐3, caspase‐8, caspase‐9, and EndoG, and their (B, C) quantification bar chart of each group are displayed. (D) Represented IHC staining images of PD‐L1 and IDO, and their (E, F) quantification bar chart of each group are displayed. (a^2^ *p*‐value <.01 vs. control; b^1^ *p*‐value <.05, b^2^ *p*‐value <.01 vs. 10 mg/kg quetiapine)

As showed in Figure 3A, the apoptosis‐related protein included caspase‐dependent and caspase‐independent molecules were all increased by quetiapine treatment. Cleaved caspase‐3, caspase‐8, and caspase‐9 that involved in extrinsic and intrinsic caspase‐dependent apoptotic pathway^22^, ^23^ were increased 1.2–1.8 times by quetiapine (Figure 3B,C). Moreover, caspase‐independent apoptosis‐related protein EndoG^24^ was also activated by quetiapine (Figure 3A‐C). We then identified whether tumor microenvironment‐associated proteins such as IDO and PD‐L1 may be altered by quetiapine. As illustrated in Figure 3D–F, though PD‐L1 may be induced by quetiapine, IDO protein which suppresses activation of cytotoxicity T cells, was suppressed by quetiapine.

### 3.4 Quetiapine did not induce tissue damage and body weight loss of Hep3B and SK‐Hep1 bearing mice

**FIGURE 4:**
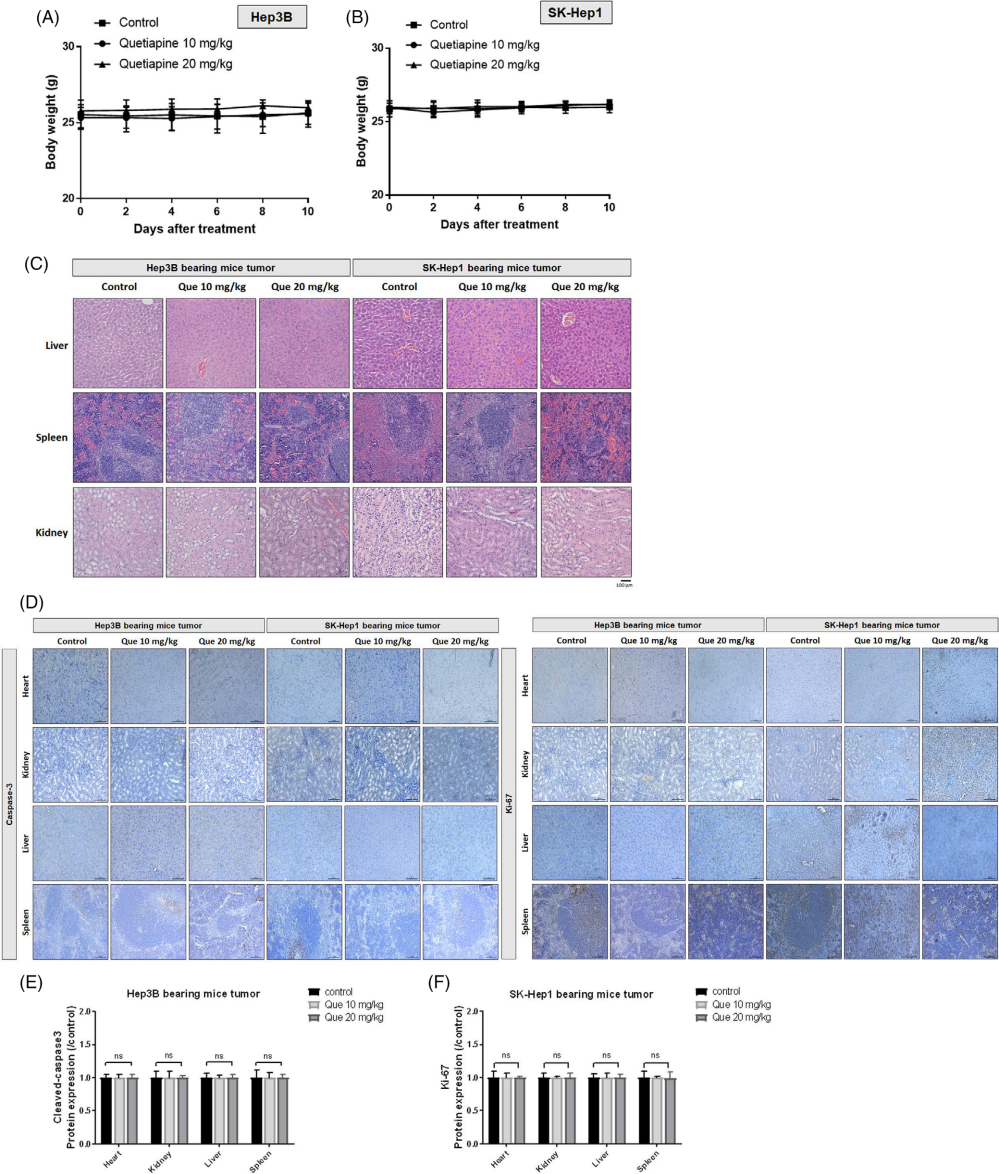
No tissue damage and body weight loss were found in quetiapine treatment under 10 and 20 mg/kg administration. (A) Hep3 B‐bearing mice and (B) SK‐Hep1‐bearing mice body weight results of each treatment group from day 0 to 10 are displayed. (C) H&E staining of liver, spleen, and kidney from Hep3B and SK‐Hep1 bearing mice are displayed. (D–F) The expression of cleaved caspase‐3 and Ki‐67 on mice liver, spleen, and kidney from Hep3B and SK‐Hep1 bearing mice are displayed. NS, no significant difference

As indicated in Figure 4A,B, the bodyweight of Hep3B and SK‐Hep1 bearing mice were not found to be altered by quetiapine treatment. In addition, the pathology pattern of liver, spleen, and kidney of each treatment group in Hep3B and SK‐Hep1 bearing mice had not been influencing by quetiapine treatment (Figure 4C). In addition, we performed cleaved caspase‐3 and ki‐67 staining on mice liver, spleen, and kidney tissue. As indicated in Figure 4D–F, no obvious induction of apoptosis and proliferation effect were found in all treatment groups. Overall results indicated that quetiapine had not induced tissue toxicity and body weight loss in HCC bearing mice.

## 4 DISCUSSION

Use of quetiapine has been found to decrease the incidence of HCC with a dose‐dependent trend when daily dose ≥30 mg.^3^ In this study, we demonstrated the inhibitory effect of quetiapine on tumor progression in HCC in vivo. In addition, we also showed a potential anti‐HCC mechanism of quetiapine.

For improvement of survival benefit of patients with HCC, more effective strategies such as immune checkpoint inhibitors therapy and tyrosine kinase inhibitors therapy have been developing.^25^ Pembrolizumab, an anti‐programmed death‐1 (PD‐1) antibody, enhances anti‐tumor cytotoxicity of T lymphocytes (CD8^+^T cells) through blockade of PD‐1 immune checkpoint signaling.^26^ HCC with positive programmed cell death ligand 1 (PD‐L1) is correlated with to pembrolizumab effect.^27^ Quetiapine significantly induced PD‐L1 expression in HCC (Figure 3D‐F) and may have the potential to increase pembrolizumab tumor response.

In addition to induction of angiogenesis, VEGF and IDO contribute to tumor progression by attenuating anti‐tumor immunity.^28^, ^29^ High VEGF expression evokes proliferation and expansion of immunosuppressive cells. Reduced VEGF level has been shown to enhance the anti‐cancer efficacy of anti‐PD‐1 antibodies.^30^ IDO mediates the conversion of tryptophan to kynurenine leading to suppression of CD8 + T cells activation.^31^ 1‐methyl‐D‐tryptophan (1‐DMT), an IDO inhibitor, has been demonstrated to boost the anti‐HCC efficacy of immune checkpoint inhibitors.^32^ Our results indicated that quetiapine effectively inhibited protein levels of both VEGF and IDO on Hep3B and SK‐Hep1 bearing animal tumor tissue (Figures 2G–I and 3D–F).

Raf/mitogen‐activated protein/ERK (MEK)/ERK is a critical oncogenic pathway to control tumor progression. ERK, a downstream component of the Raf/MEK/ERK pathway, activates downstream kinases and transcription factors resulting in the expression of proliferation, survival, angiogenesis, and invasion‐associated proteins.^33^, ^34^ NF‐κB, a transcription factor of numerous oncogenes, participates in the expression of ERK‐mediated downstream effector proteins.^35^, ^36^ Sorafenib and regorafenib are oral multi‐tyrosine kinase inhibitors used to treat patients with HCC. Our previous studies presented that inhibition of ERK/NF‐κB signaling‐mediated proliferation, anti‐apoptotic, and invasion‐associated proteins expression was associated with both sorafenib and regorafenib‐inhibited progression of HCC.^36^, ^37^

Our data presented that quetiapine effectively inhibited the growth of HCC in vivo (Figure 1). Furthermore, liver, kidney, spleen pathology alteration, and general toxicity were not induced by quetiapine treatment on Hep3B and SK‐Hep1 bearing animals (Figure 4). We also found protein levels of NF‐κB p65 (Sre536), ERK1/2 (Thr202, Tyr204), MCL‐1, C‐FLIP, XIAP, Cyclin‐D1, and MMP‐9 were significantly reduced by quetiapine treatment (Figure 2A–F). Inhibition of anti‐apoptotic proteins and induction of apoptosis by complementary agents have been presented to boost the anti‐HCC efficacy of therapeutic agents.^38^, ^39^ In addition to the reduction of anti‐apoptotic proteins, expression of apoptotic proteins cleaved‐caspase‐3, −8, −9, and Endo‐G was significantly increased by quetiapine treatment (Figure 3A‐C). Endo‐G is a caspase‐independent death effector to trigger nucleosome DNA fragmentation leading to tumor cell death.^40^ Expression of Endo‐G was deceased in human HCC tissues.^41^ Our results in figure 3A‐C indicated that quetiapine may induce Endo‐G expression in HCC bearing mice tumor.

In conclusion, suppression of ERK/NF‐κB signaling and induction of apoptosis through extrinsic/intrinsic caspase‐dependent and caspase‐independent pathways are involved in the quetiapine‐inhibited progression of HCC in vivo. According to the therapeutic efficacy and potential mechanism, we suggest that quetiapine as a complementary agent may provide therapeutic benefits to patients with HCC. Further clinical trial is warranty on the base of our preclinical and published retrospective national health database studies.

## CONFLICT OF INTEREST

The authors do not have any conflicts of interest to disclose.

## Data Availability

The data that support the findings of this study are available on request from the corresponding author.
